# Sentinel fracture: the necessity of improved post-fracture care

**DOI:** 10.1007/s10354-024-01066-4

**Published:** 2024-11-29

**Authors:** Katharina Kerschan-Schindl, Harald Widhalm, Anna Pataraia, Peter Nicolakis, Martin Frossard, Mohammad Keilani, Michael Mickel, Stefan Hajdu, Richard Crevenna

**Affiliations:** 1 Department of Physical Medicine, Rehabilitation and Occupational Medicine, Medical University of Vienna/Vienna General Hospital, Vienna, Austria; 2 Department of Orthopedics and Trauma-Surgery, Clinical Division of Traumatology, Medical University of Vienna/Vienna General Hospital, Vienna, Austria

**Keywords:** Osteoporosis, Sentinel fracture, Refracture risk stratification, Fracture liaison service, Osteoporose, Sentinelfraktur, Frakturrisikostratifizierung, Fracture Liaison Service

## Abstract

Fragility fractures caused by osteoporosis, the most common metabolic bone disease, place a significant burden on affected individuals and impose substantial economic costs. A fragility fracture implies an imminent elevated risk for subsequent fractures, particularly in the short term. Therefore, osteoporosis must be addressed in the event of a sentinel fracture, if not already previously treated. Regardless of whether the fracture is treated conservatively or surgically, post-fracture care is particularly important. Early mobilization followed by fall risk assessment and the initiation of adequate bone-specific medication are essential milestones in preventing subsequent fractures. Monitoring patients increases adherence to bone-specific medication and fall prevention strategies. Comprehensive post-fracture care is important and should be performed by a multidisciplinary team. Coordinated care models, such as the fracture liaison service (FLS), have shown enhancements in the initiation of and adherence to secondary prevention of fragility fractures. Despite recommendations by several guidelines including that published by the Austrian Society for Bone and Mineral Research, only one fracture liaison service has been implemented in Austria.

## Abstract (de)

Durch Osteoporose, häufigste Erkrankung des Knochenstoffwechsels, bedingte Fragilitätsfrakturen stellen sowohl für die Betroffenen als auch für das Gesundheitssystem eine enorme Belastung dar. Jede Fraktur bedeutet speziell im ersten Jahr danach ein stark erhöhtes Risiko für weitere Frakturen. Aus diesem Grund müssen wir im Fall einer Sentinelfraktur – sofern nicht schon vorher geschehen – die Osteoporose behandeln. Unabhängig davon, ob die Patienten konservativ oder chirurgisch behandelt werden, ist die Nachbehandlung der Fraktur wichtig. Eine frühzeitige Mobilisierung, gefolgt von einem Sturzrisikoassessment und der Einleitung einer knochenspezifischen Medikation sind wesentlich zur Reduktion des Risikos neuerlicher Fakturen. Werden die Betroffenen einige Wochen und ein Jahr nach der Fraktur nochmals von einem Osteoporosespezialisten gesehen, erhöht dies die Compliance der Patientinnen/Patienten. Im Optimalfall wird ein multimodales Management nach einer Fraktur von einem multidisziplinären Team durchgeführt. Obwohl koordinierte Versorgungsmodelle, wie das Fracture Liaison Service (FLS) in etlichen Leitlinien empfohlen werden, darunter auch in der von der Österreichischen Gesellschaft für Knochen- und Mineralforschung veröffentlichten Leitlinie, wurde in Österreich bisher nur ein FLS implementiert.

An 87-year-old woman is brought to the emergency department by ambulance after falling at her retirement home. She reports pain in the region of her left hip. Her left leg is visibly shortened, rotated outward, and she is unable to lift it. The X‑ray shows a fracture line in the area of the medial femoral neck. The patient undergoes dynamic hip screw placement on the same day, and mobilization begins the following day. Based on the medical history, the patient had previously suffered several fractures—a radius fracture, a pelvic fracture and a fracture of the femoral neck on the opposite side. How do you plan to assess this patient and reduce the risk of future fractures?

## The challenge

Osteoporosis is a skeletal disorder characterized by compromised bone strength; it is influenced by bone density and bone quality and predisposes a person to an increased risk of fracture [1]. Osteoporosis is a global health problem, with about 200 million people affected [2]. According to the Scorecard for Osteoporosis in Europe (SCOPE), which summarizes key indicators of the burden of osteoporosis and its management, 32 million subjects were estimated to have osteoporosis in the 27 countries of the European Union plus Switzerland and the United Kingdom (EU 27 + 2), and 4.3 million new fragility fractures were sustained in 2019 [3]. In Austria in 2019, the estimated prevalence amounted to 552,000 osteoporotic subjects (according to the definition of the World Health Organization) and 110,000 new fragility fractures, equivalent to almost 500 fractures per hour or eight factures per minute [4]. Concerning the annual incidence of hip fractures in women from European countries, Austria is among the top three [3]. The challenge is escalating, as osteoporosis and related fragility fractures are anticipated to rise significantly in the coming years.

### Social and economic burden

Fractures have a major impact on quality of life. They do not only cause pain and disability in the short term but may also be followed by loss of autonomy or even premature mortality. In terms of their impact on an individual’s health, hip fractures are particularly devastating: 1‑year mortality after hip fracture is supposed to be between 20% and 30% [5]. However, excess mortality is elevated even over the subsequent two decades, with cardiovascular disease and pneumonia being the most frequent life-threatening conditions [6]. In those patients who survive, hip fracture has a major impact on care status. A study based on Swedish population registers showed that two thirds of hip fracture patients receive municipal care 3 months after the event compared to half of health-matched controls [7]. The rate of wheelchair use following hip fracture far exceeds that of the general population [8]. Psychosocial symptoms, most notably depression and loss of self-esteem, may be additional consequences of those affected. Osteoporotic fractures cause a higher loss of disability-adjusted life years (DALYs) than stroke or chronic obstructive pulmonary disease [9] and a higher loss than most common cancers [10]. According to a pooled analysis of prospective cohorts involving over 220,000 participants (none of whom had experienced a previous hip fracture) followed for more than 13 years, hip fractures result in an average reduction of 2.7% in healthy life expectancy [11].

In addition to this substantial human impact, osteoporosis and associated fragility fractures also account for an enormous economic cost. In the EU 27 + 2, incident fracture costs, intervention costs, and long-term disability costs amounted to almost € 57 billion in 2019 [3]. In Austria, for the same year, the direct cost of incident fractures and the ongoing cost of previous fractures amounted to 3.4% of all healthcare expenditure—€ 1.3 billion. Assessment and pharmacological therapy only accounted for a very small proportion of the total sum—€ 41 million [4]. The average direct cost of osteoporotic fractures for each individual in Austria and the EU27 + 2 was similar. Frail patients with hip or pelvic fractures who require extended hospital stays and subsequent admission to a nursing home generate particularly high costs. From an economic point of view, fragility fractures have a notable impact on the healthcare budget in EU27 + 2 as well as in Austria.

### Imminent fracture risk

The risk of a subsequent fracture is highest immediately after the initial fracture. According to a cross-sectional population-based study of postmenopausal women, compared with the risk of a first fracture, the relative risk (RR) of a subsequent fracture was as high as 5.3 in year one after the sentinel fracture [12]. The re-fracture risk depends on age, gender, and the sentinel fracture. A 70-year-old woman, for instance, has the highest re-fracture risk after a sentinel spine fracture (HR 6.6) and the lowest re-fracture risk after a sentinel forearm fracture (HR 3.5) [13]. The risk of a new major osteoporotic fracture (MOF; i.e., hip fracture, clinical vertebral fracture, forearm fracture, humerus fracture) decreases with increasing time, but always remains higher than for someone who has never suffered an MOF [14]. The timing of the re-fracture risk depends on the sentinel fracture as well. Following a sentinel hip fracture, 45% of those patients who experience a subsequent fracture do so within 1 year; the corresponding percentages for spine, forearm, and humeral fractures are 42%, 31%, and 41%, respectively [13]. The occurrence of a vertebral fracture increases not only the risk of a further vertebral fracture but of a non-vertebral fracture as well, and the higher the number of prevalent vertebral fractures, the higher the risk [15]. On the other hand, the risk of vertebral fractures and hip fractures is increased independently of the index fracture site [16].

Altogether, any sentinel fracture increases the likelihood of any subsequent fracture—particularly immediately after the fracture occurs. Therefore, identification of patients with sentinel fracture, accurate diagnosis of osteoporosis, and adequate treatment are indispensable in post-fracture care.

## Strategies

### Evaluation

A study in eight countries across Europe showed that 75% of elderly women seen in primary care, who are at a high risk of a fragility fracture, have no specific therapy; in those without the diagnosis, this percentage exceeds 90%. Thus, we have a large treatment gap in Europe [17]. Even in the case of a prevalent fragility fracture, a very low number of patients are assessed and adequately treated for osteoporosis. In Austria, only one in ten men and less than two in ten women receive specific treatment after their osteoporotic fracture [18]. Even more dramatic is that only 14% of patients experiencing a hip fracture receive osteoporotic treatment afterwards [19]. The step before starting treatment is identifying patients with osteoporosis, with osteoporotic fractures.

Given the very high risk of re-fracture following a sentinel fracture, we must try hard to ensure that the first fracture is the last. This involves enhancing post-fracture care in addition to surgical treatment. It is essential to identify patients who have experienced any fracture that may indicate osteoporosis. Understaffing and lack of time is of course a major problem in patient care. Nevertheless, we have to take the time to inquire about the circumstances of the accident. This is the key to deciding whether adequate trauma is responsible for the fracture. If patients have experienced a relatively trivial traumatic event or cannot recall any antecedent trauma, a bone mineral density (BMD) measurement, a key component for the management of osteoporosis, should be performed. According to the DVO (*Dachverband Osteologie*), an umbrella organization for osteology, bone-specific medication should be initiated without waiting for a BMD measurement in the case of multiple low-trauma vertebral fractures with at least 20% height loss, a single low-trauma vertebral fracture with at least 25% height reduction, or a hip fracture; in this, case bone-specific medication should be initiated independently of bone mineral density (https://leitlinien.dv-osteologie.org/—accessed 2024.06.18). The Austrian osteoporosis guideline recommends immediate initiation of bone-specific therapy after vertebral or hip fracture as well [20]. Biochemical analysis, however, is essential to rule out secondary causes of bone loss and/or fractures as well as contraindications to drugs. The fracture risk can be estimated using the web-based risk assessment model for fractures FRAX (https://frax.shef.ac.uk/frax/).

### Management

#### Mobilization

The first goal of acute fracture treatment includes effective pain management and prompt mobilization. When using a plaster cast or plastic splint—common treatments for distal radius fracture—adjacent joints should be used in daily activities. In the case of a fracture of the proximal humerus treated with a shoulder bandage, the European Society for Trauma and Emergency Surgery (ESTES) recommends starting pendulum exercises and guided movement exercises up to 90 degrees after 3 weeks [21]. Surgically treated fractures are usually immobilized in a shoulder bandage for 2–4 weeks, usually with passive exercise (swinging) after 2 weeks and active exercise after 4 weeks [22]. After surgery, fractures near the hip are usually exercise stable [23]. Ideally, the patient should be transferred directly to a remobilization facility to swiftly regain their pre-fracture level of activities of daily living (ADL). Multidisciplinary rehabilitation, particularly with the inclusion of progressive strength training (inpatient/outpatient) for fractures close to the hip joint, reduces mortality and improves mobility [24, 25]. In accordance with the international consensus on the management of vertebral fractures, an individualized guided exercise program should be initiated when the pain level decreases or after medical clearance (around 4–12 weeks after the vertebral fracture) [26]. An exercise program supervised by a physiotherapist improves pain and function [27]. However, if pain persists, it is recommended to perform the exercises for the back extensors in a relieved position (supine position) [28]. Fracture healing (around the 12th week after fracture) is the time to initiate a multimodal training program that includes progressive strength training, functional training, and balance training [26]. In general, lifting heavy loads is prohibited for 12 weeks in the case of vertebral body fractures, and in the case of pronounced osteoporosis it is even strictly prohibited. Kyphoplasty/vertebroplasty procedures, either alone or combined with dorsal instrumentation, generally offer stable postsurgical weightbearing, depending on pain management [29]. In general, spinal orthoses should not be routinely prescribed but rather considered on an individual basis considering the pain situation, with acute pain being the primary factor for prescription [29, 30]. Orthoses can be used as a pain-relieving measure to improve pain and trunk muscle strength by wearing orthoses (2 h a day for 6 months) [28]. Patients with vertebral fractures and/or multiple fragility fractures are generally advised to avoid high-impact loads on the spine (above the level of everyday stress, e.g., higher impact loads than during brisk walking) [31].

#### Osteoporosis treatment (according to the Austrian osteoporosis guideline [20])

The aim of any osteoporosis therapy is to prevent the first fracture or at least a further fracture. All patients after the age of 50 with an MOF must be assessed for the initiation of treatment. Does it make sense to prescribe a bone-specific medication in the acute setting within a few weeks after a fracture? There have been repeated concerns that osteoporosis treatment could impede fracture healing. However, some evidence exists from both preclinical and clinical studies that teriparatide accelerates fracture healing, that function is largely improved, and that pain is relieved [32]. Both the physiological processes of fracture healing and experimental investigations point toward enhanced fracture healing through administration of the sclerostin antibody romosozumab. However, this could not be verified in a clinical, albeit small, study [33]. Gao and coauthors [34] concluded in their meta-analysis that bisphosphonates do not influence fracture healing. A subgroup analysis of the fractures occurring in the pivotal study of the receptor activator nuclear factor B ligand (RANKL) antibody denosumab has shown that there is definitely no delayed healing in these patients [35]. Therefore, if necessary, bone-specific medication should be started as soon as possible.

The administration of calcium (1000 mg) and/or vitamin D (800 IU) is the basis of any osteoporosis treatment and is necessary as a supplement to the drug treatment of osteoporosis if the calcium intake with nutrition is low or if there is a vitamin D insufficiency. A vitamin D deficiency/insufficiency must be compensated before starting any osteoporosis-specific treatment. An adequate protein supply is also very important. The German Nutrition Society recommends a daily protein intake of at least 0.8 g/kg bodyweight (target weight) and 1.0 g/kg bodyweight for the age group 65+ [36].

Drugs used in osteoporosis treatment can be divided into two broad categories depending on their primary mode of action: antiresorptive drugs primarily inhibit osteoclastic bone resorption, with subsequent secondary effects on bone formation. Osteoanabolic drugs primarily stimulate bone formation via increased osteoblast activity, with various effects on bone resorption. Anabolic drugs lead to a rapid improvement in bone microarchitecture and subsequently to an improvement in mineralization. Romosozumab, however, has a dual action by stimulating bone formation and inhibiting bone resorption, which also leads to a rapid improvement in microarchitecture and mineralization.

Assessment of the 10-year fracture probability for an MOF and the isolated near-hip fracture using the Austria-specific FRAX® version enables grading of the individual fracture risk into a low, high, or very high fracture risk [37]. Individuals with a low risk of fracture should receive advice on adopting a “bone-protective” lifestyle (diet, exercise, nicotine abstinence, low alcohol consumption). Patients with a high fracture risk without prevalent fractures should primarily be treated with antiresorptive substances. Men and women with a very high fracture risk should preferably be given osteoanabolic therapy by a specialist with knowledge and experience in the field of osteoporosis. The presence of a recent (more recent than 24 months) MOF is the corresponding indication. In case of contraindications to first-line therapy, anabolic drugs, or medication with a dual mode of action, antiresorptive therapy represents an alternative treatment option. In the event of a hip fracture, zoledronate should be administered at the earliest 14 days afterwards; denosumab can be administered immediately. Of course, this applies only in the absence of contraindications. The choice of medication depends on any existing contraindications, the patients’ health and mental state, and their compliance. Additionally, it is important to plan a long-term treatment strategy for each patient before starting osteoporosis-specific treatment, as the timing and sequencing of the use of certain drugs is important.

To meet the requirements of the Austrian osteoporosis guidelines, all patients over the age of 50 with an MOF (hip, spine, humerus, or forearm) should be investigated with regard to the initiation of treatment.

#### Fall risk prevention

The risk of falling increases with age. Looking at the 65+ age group, one third fall at least once a year [38]. In the 80+ age group, the frequency is even higher. If the impact of a fall—depending on impact location, soft tissue attenuation, muscle activity, and the surface—is high, the structural capacity may not be able to withstand the applied loads, leading to fractures. Not vertebral fractures, but most fractures occur because of a fall, defined as an “unexpected event in which the subject comes to rest on the ground, floor, or lower level” [39]. Regardless of fracture occurrence, a recent fall (within the previous 4 months) indicates an imminent risk of fracture in the following year [40]. This increase in fracture risk is largely independent of BMD [41]. Hence, fall prevention is important.

Regarding the identification of older adults at a high risk of falling, a distinction needs to be made between opportunistic case finding in clinical practice and assessing patients who have experienced a fall or fall-related injury. For a subject aged 65 years or older without a fall-related injury in the preceding year, the Task Force on Global Guidelines for Falls in Older Adults recommends gait speed or, alternatively, the timed up and go test for fall prediction [42]. Most often, both external and internal factors, such as balance deficits, contribute to the onset of falls [43]. We should ask older adults presenting with a fall-related injury about the details of the event. In subjects with a recent fragility fracture, a multifactorial assessment may be indispensable.

Since most falls are of multifactorial origin, it is often necessary to address numerous modifiable risk factors associated with falls. A Cochrane Review showed that multifactorial intervention following assessment of the participants’ risk profile reduced the rate of falls by 23%. Besides medication review, interventions included psychological interventions, environment or assistive technologies, and/or exercise [44]. Exercise as a single intervention reduces the rate of falls by 23%; looking at different exercise modalities, balance and functional exercises reduce the rate of falls by 24%, combined with resistance training they are even more effective [45]. According to the world guidelines for fall prevention, especially tailored exercises for balance, gait, and strength are an essential part of fall prevention [42].

Therefore, it is crucial to focus on balance training and functional exercises following immediate post-fracture mobilization. Instructions on how to perform the exercises correctly and safety during the exercises are vital. This is especially important in frail subjects with a recent fragility fracture. There are some aspects worth mentioning: balance training is most effective in persons with a high risk of falling [46]; however, these effects generally do not endure beyond the completion of training. After a fall at home leading to a fragility fracture, it additionally makes sense to remove tripping hazards like carpets. Home fall-hazard interventions are most effective when targeted to people at a higher risk of falling ([47]).

## The post-fracture care program fracture liaison service (FLS)

Post-fracture care programs managing patients with a fragility fracture aim to reduce subsequent fractures. One of the most common coordinator-based post-fracture care programs is the fracture liaison service (FLS). The framework consists of four steps: 1) identification of suitable patients, 2) assessment including fracture risk and potential secondary causes of osteoporosis, 3) personalized treatment recommendation, and 4) short- and long-term follow-ups in order to improve adherence to treatment [48]. Eleven key performance indicators have to be completed within certain timespans after fracture [49]. FLSs improve treatment rates and adherence to treatment, and they reduce the risk of re-fracture and mortality [50–52]. Concerning the economic aspect, FLSs are cost effective or even cost saving [50]. According to a recent review including 23 FLSs, 86% of FLSs have a positive return on investment, with more intensive FLSs showing a higher return on investment than non-intensive FLSs [53].

European as well as US clinical guidelines for the prevention and treatment of osteoporosis recommend multidisciplinary coordinator-based FLS in post-fracture care [54–56]. Up to now, 929 FLSs are registered in all regions of the world (https://www.osteoporosis.foundation/our-network—assessed 2024.08.05). Despite the fact that the DVO guideline (https://leitlinien.dv-osteologie.org/) and the Austrian guideline recently published by the Austrian Society of Bone and Mineral Research [20] also recommend coordinated care models such as the FLS, so far only one FLS has been implemented in Austria [57]. Thus, there is much room for improvement.

Clinical key points of post fracture care and secondary fracture prevention are summarized in Table 1.

**Table 1:** Key clinical points

– A recent fragility fracture implies an imminent risk of a subsequent fracture
– Clarifying details about the trauma/fall leading to the fracture is necessary
– Mobilization/rehabilitation is recommended as soon the fracture is exercise stable
– Sufficient intake/supplementation of proteins, calcium, and vitamin D is necessary for muscle and bone metabolism
– Bone-specific medication does not compromise fracture healing
– Bone mineral density measurement is not always necessary before starting osteoporosis treatment
– Biochemical analyses are obligatory before treatment initiation
– The choice of bone-specific medication depends on fracture risk
– Follow-up examinations improve adherence to treatment
– Multifactorial intervention based on fall risk assessment is effective in reducing the risk of falls
– Comprehensive care for fracture patients is best embedded in a post-fracture care program like the fracture liaison service
– Fracture liaison services reduce the re-fracture risk and are cost effective

## Conclusion and recommendations

The 87-year-old woman described at the beginning of this article is at a high risk of another re-fracture, given the recurrent falls that have already led to multiple fractures. She definitely requires optimal post-fracture care. A low calcium nutritional intake and low serum levels of vitamin D represent the rationale for supplementation with calcium and vitamin D. If no other reason for the fracture can be detected through history or blood tests, bone-specific medication must be prescribed as soon as serum levels of albumin-corrected calcium and vitamin D are within the normal range—preferably osteoanabolic or dual-action therapy. Optimal post-fracture care also includes BMD measurement and fall risk prevention after specific assessment. A follow-up examination should be scheduled a few weeks and 1 year after the fracture, with the aim of improving compliance with drug therapy and fall-prevention strategies.
